# Emergency Department Visits for Firearm Injuries Before and During the COVID-19 Pandemic — United States, January 2019–December 2022

**DOI:** 10.15585/mmwr.mm7213a2

**Published:** 2023-03-31

**Authors:** Marissa L. Zwald, Miriam E. Van Dyke, May S. Chen, Lakshmi Radhakrishnan, Kristin M. Holland, Thomas R. Simon, Linda L. Dahlberg, Norah W. Friar, Michael Sheppard, Aaron Kite-Powell, James A. Mercy

**Affiliations:** ^1^Division of Violence Prevention, National Center for Injury Prevention and Control, CDC; ^2^Epidemic Intelligence Service, CDC; ^3^Office of Public Health Data, Surveillance, and Technology, CDC.

During the COVID-19 pandemic, the U.S. firearm homicide rate increased by nearly 35%, and the firearm suicide rate remained high during 2019–2020 (*1*). Provisional mortality data from the National Vital Statistics System indicate that rates continued to increase in 2021: the rates of firearm homicide and firearm suicide in 2021 were the highest recorded since 1993 and 1990, respectively (*2*). Firearm injuries treated in emergency departments (EDs), the primary setting for the immediate medical treatment of such injuries, gradually increased during 2018–2019 (*3*); however, more recent patterns of ED visits for firearm injuries, particularly during the COVID-19 pandemic, are unknown. Using data from the National Syndromic Surveillance Program (NSSP),* CDC examined changes in ED visits for initial firearm injury encounters during January 2019–December 2022, by year, patient sex, and age group. Increases in the overall weekly number of firearm injury ED visits were detected at certain periods during the COVID-19 pandemic. One such period during which there was a gradual increase was March 2020, which coincided with both the declaration of COVID-19 as a national emergency^†^ and a pronounced decrease in the total number of ED visits. Another increase in firearm injury ED visits occurred in late May 2020, concurrent with a period marked by public outcry related to social injustice and structural racism (*4*), changes in state-level COVID-19–specific prevention strategies,^§^ decreased engagement in COVID-19 mitigation behaviors (*5*), and reported increases in some types of crime (*4*). Compared with 2019, the average number of weekly ED visits for firearm injury was 37% higher in 2020, 36% higher in 2021, and 20% higher in 2022. A comprehensive approach is needed to prevent and respond to firearm injuries in communities, including strategies that engage community and street outreach programs, implement hospital-based violence prevention programs, improve community physical environments, enhance secure storage of firearms, and strengthen social and economic supports.

CDC used near real-time electronic health record data from NSSP to examine changes in ED visits for initial firearm injury encounters during the COVID-19 pandemic. Temporal trends were assessed for three surveillance periods (calendar years 2020, 2021, and 2022) and compared with visits from calendar year 2019. Only facilities consistently reporting more complete data^¶^ during 2019–2022 were included. Firearm injury ED visits were identified using a categorization including administrative diagnosis codes and free-text reason-for-visit (chief complaint terms), developed and validated by CDC in partnership with state, tribal, local, and territorial health departments** (Supplementary Table, https://stacks.cdc.gov/view/cdc/125985). The mean number of weekly ED visits for firearm injuries, percent change in mean weekly ED visits for firearm injuries,^††^ and visit ratios (VRs)^§§^ with 95% CIs were examined overall, and by age group (0–14, 15–24, 25–34, 35–64, and ≥65 years) for females and males. All analyses were conducted using R software (version 4.1.2; R Foundation). This activity was reviewed by CDC and was conducted consistent with applicable federal law and CDC policy.^¶¶^

Coinciding with the declaration of COVID-19 as a national emergency on March 13, 2020, the weekly number of firearm injury ED visits began to increase, despite a steep decline in the total number of ED visits (Figure). The weekly number of firearm injury ED visits also sharply increased during the week of May 24, 2020, and remained high for the rest of 2020. Trends were similar among females and males.

**FIGURE:**
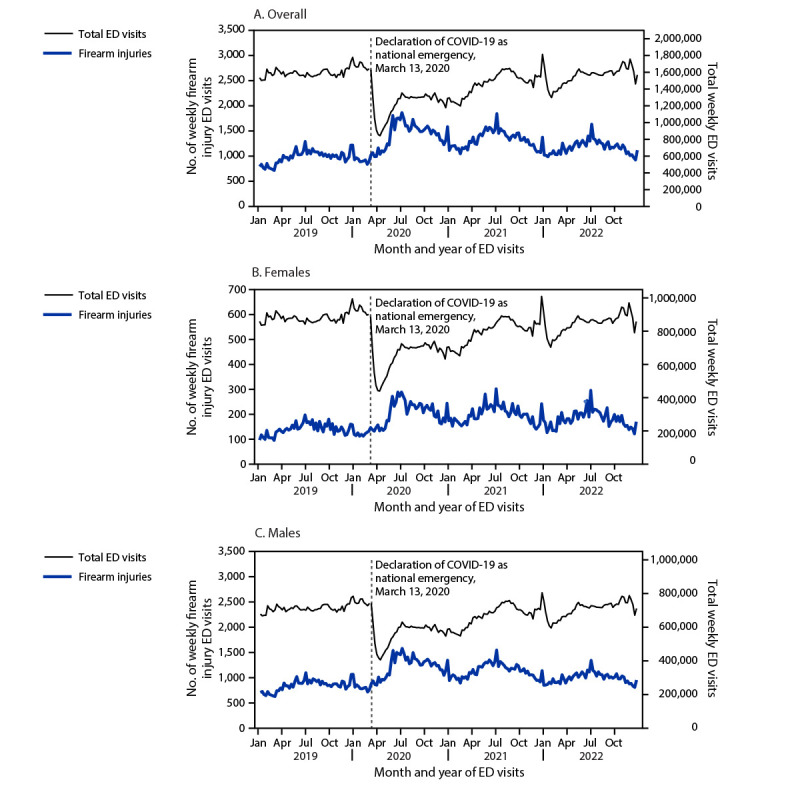
Weekly number of emergency department visits for firearm injury,* overall (A) and among females (B) and males† (C) — National Syndromic Surveillance Program,^§^ United States, January 2019–December 2022^¶^ **Abbreviations:** ED = emergency department; NSSP = National Syndromic Surveillance Program. * ED visits for an initial firearm injury encounter were identified by querying a categorization developed and validated by CDC in partnership with state, tribal, local, and territorial health departments. The following intent types were included in the definition: unintentional, intentional self-directed, assault, undetermined intent, legal intervention, and terrorism. ^†^ The y-axis scales differ among overall, female, and male figure panels. ^§^ NSSP is a collaboration among CDC, local and state health departments, and federal, academic, and private sector partners. NSSP receives medical record data from approximately 75% of EDs nationwide, although fewer than 50% of facilities from California, Hawaii, Minnesota, and Oklahoma currently participate in NSSP. https://www.cdc.gov/nssp/index.html ^¶^ Data through December 2022 are included even though November and December are not included on the x-axes.

During the study period, compared with 2019, mean weekly ED visits for firearm injury were 37% higher in 2020, 36% higher in 2021, and 20% higher in 2022, with differences by sex-specific age group (Table). Among both females and males, mean weekly ED visits for firearm injuries were consistently highest among persons aged 15–24 years across the entire study period. However, the largest increases in the proportion of firearm injury ED visits were among persons aged 0–14 years during 2020 (VRs = 2.81 for females and 2.31 for males, respectively), 2021 (VRs = 2.20 and 1.85), and 2022 (VRs = 1.49 and 1.44), compared with 2019.

**TABLE:** Mean weekly number of emergency department visits, percent change* in emergency department visits, and visit ratios† of emergency department visits for firearm injury,^§^ overall and by sex and age group — National Syndromic Surveillance Program,^¶^ United States, January 2019–December 2022

Sex/Age group, yrs	2019	2020**			2021**			2022**		
	Mean weekly no. of firearm injury ED visits	Mean weekly no. of firearm injury ED visits	% Change in mean weekly no. of firearm injury ED visits	VR (95% CI)	Mean weekly no. of firearm injury ED visits	% Change in mean weekly no. of firearm injury ED visits	VR (95% CI)	Mean weekly no. of firearm injury ED visits	% Change in mean weekly no. of firearm injury ED visits	VR (95% CI)
**All**	**979.3**	**1,341.5**	**37.0**	**1.66 (1.64–1.68)**	**1,328.3**	**35.6**	**1.46 (1.44–1.48)**	**1,170.0**	**19.5**	**1.22 (1.20–1.23)**
**Females**										
Overall	**139.6**	**190.9**	**36.7**	**1.70 (1.64–1.75)**	**198.0**	**41.9**	**1.55 (1.51–1.60)**	**179.7**	**28.7**	**1.33 (1.29–1.37)**
0–14	6.7	11.3	69.3	2.81 (2.47–3.21)	11.4	71.1	2.20 (1.93–2.52)	9.5	43.1	1.49 (1.30–1.71)
15–24	45.8	64.5	40.8	1.76 (1.67–1.86)	66.4	45.0	1.60 (1.52–1.69)	62.1	35.5	1.47 (1.40–1.55)
25–34	38.2	54.5	42.6	1.71 (1.62–1.81)	55.5	45.2	1.59 (1.50–1.68)	49.6	29.7	1.41 (1.33–1.49)
35–64	41.5	50.7	22.2	1.45 (1.37–1.53)	54.5	31.4	1.40 (1.33–1.49)	51.0	22.9	1.29 (1.22–1.36)
≥65	7.5	10.0	33.7	1.57 (1.38–1.79)	10.2	37.0	1.42 (1.24–1.62)	7.6	1.0	0.96 (0.83–1.10)
**Males**										
Overall	**839.8**	**1,150.6**	**37.0**	**1.62 (1.60–1.64)**	**1,130.3**	**34.6**	**1.42 (1.41–1.44)**	**990.3**	**17.9**	**1.18 (1.17–1.20)**
0–14	22.1	30.1	36.1	2.31 (2.14–2.49)	31.8	43.7	1.85 (1.72–2.00)	30.9	39.9	1.44 (1.33–1.55)
15–24	291.9	404.6	38.6	1.67 (1.64–1.70)	383.7	31.4	1.42 (1.39–1.45)	343.8	17.7	1.24 (1.21–1.27)
25–34	250.2	351.1	40.3	1.55 (1.51–1.58)	339.2	35.6	1.40 (1.37–1.43)	286.6	14.6	1.21 (1.18–1.24)
35–64	222.3	297.5	33.8	1.47 (1.43–1.50)	310.7	39.8	1.42 (1.38–1.45)	287.2	29.2	1.31 (1.28–1.35)
≥65	53.2	67.3	26.4	1.38 (1.31–1.45)	64.9	21.9	1.20 (1.14–1.26)	41.8	-21.5	0.72 (0.68–0.76)

**Abbreviations**: ED = emergency department; NSSP = National Syndromic Surveillance Program; VR = visit ratio. * Percent change in visits per week during each surveillance period was calculated as ([mean weekly ED visits for firearm injury during surveillance period − mean weekly ED visits for firearm injury during comparison period] / mean weekly ED visits for firearm injury during comparison period) × 100.

^†^ VR = (ED visits for firearm injury [surveillance period] / all ED visits [surveillance period]) / (ED visits for firearm injury [comparison period] / all ED visits [comparison period]). Ratios >1 indicate a higher proportion of ED visits for firearm injury during the surveillance period than the comparison period; ratios <1 indicate a lower proportion during the comparison period than during the surveillance period; 95% CIs that do not include 1 were considered statistically significant.

^§^ ED visits for an initial firearm injury encounter were identified by querying a categorization developed and validated by CDC in partnership with state, tribal, local, and territorial health departments. The following intent types were included in the definition: unintentional, intentional self-directed, assault, undetermined intent, legal intervention, and terrorism.

^¶^ NSSP is a collaboration among CDC, local and state health departments, and federal, academic, and private sector partners. NSSP receives medical record data from approximately 75% of EDs nationwide, although fewer than 50% of facilities from California, Hawaii, Minnesota, and Oklahoma currently participate in NSSP. https://www.cdc.gov/nssp/index.html

** Comparison period is calendar weeks 1–52 (December 30, 2018–December 28, 2019).

## Discussion

Increases in firearm injury ED visits were detected at certain periods during the COVID-19 pandemic. Beginning the week of March 13, 2020, concurrent with the declaration of COVID-19 as a national emergency,^†^ implementation of community mitigation measures, and a decline in ED visits overall, the weekly number of firearm injury ED visits began to increase. A sharp increase in the weekly number of firearm injury ED visits occurred beginning the week of May 24, 2020, which remained elevated throughout 2020. Although this report did not assess causes for the observed increases or differentiate by intent type, the rise in visits in late May 2020 corresponded with a period of increased social unrest over strained law enforcement–community relations and longstanding systemic inequities, structural racism, and trauma experienced by racial and ethnic minority groups in the United States*** (*4*,*6*); changes in the implementation of and engagement in COVID-19 mitigation measures (*5*); and reported increases in some types of crime (*4*). Additional research is needed to better understand recent trends in firearm injuries by intent, sociodemographic characteristics, and contextual factors to help guide tailored prevention efforts and address inequities in the risk for firearm injuries (*1*).

The overall increases in firearm injury ED visits during the COVID-19 pandemic highlighted in this study are consistent with previous research that indicated increasing national rates of firearm violence during the COVID-19 pandemic (*7*) and increases in the number of pediatric ED visits for firearm injuries during 2020 and 2021 compared with 2019 (*8*). The mean weekly number and proportion of firearm injury ED visits were higher during 2020, 2021, and 2022 compared with 2019. These patterns were observed for both sexes and across most age groups, with the youngest age group (0–14 years) experiencing the largest increase in the proportion of firearm injury ED visits. Challenges faced by children and adolescents during the COVID-19 pandemic might have influenced their risk for firearm injury, including disruptions to daily routines and schooling (e.g., social isolation, physical distancing, and increased time spent at home, potentially increasing access to firearms in the home); changes in health care access (e.g., limited access to mental health services); and diminished security and safety (e.g., housing and financial insecurity, increased exposure to violence, threat of illness, and uncertainty about the future).^†††^ Previous studies have also cited increases in firearm purchases and limited parental supervision as potential factors associated with heightened risk for firearm injuries among children and adolescents during the COVID-19 pandemic (*9*,*10*).

The findings in this study are subject to at least six limitations. First, NSSP ED visit data are collected using a convenience sample with geographic variations in coverage; findings are not generalizable to nonparticipating facilities and allow for limited inference regarding the underlying prevalence of firearm injuries outside EDs. However, NSSP ED visit data represent approximately 75% of U.S. EDs^§§§^; national trends observed in this analysis are likely representative of firearm injuries resulting in emergency care during the pandemic. Second, variations in data quality and coding practices might over- or underestimate visit trends. To address this, data were only analyzed from facilities with consistent reporting during the period of study, and the number of these EDs remained relatively constant over time. Third, the categorization used in this study captures firearm injuries overall and does not differentiate by intent. Fourth, the cross-sectional nature of this analysis, which uses electronic health record data, does not allow for causal inferences regarding changes in visit trends or contributing factors. Fifth, this study assessed changes in firearm injury ED visit counts rather than rates, which have been difficult to interpret during the COVID-19 pandemic because of changes in ED utilization that substantially affected the denominator typically used to calculate ED visit rates; still, the large number of weekly firearm injury ED visits, broad NSSP coverage, and 4-year study period allowed for a robust analysis of trends over time. Finally, some patient demographic information and facility characteristics were unavailable or incomplete at the aggregated national level. Future and ongoing collaborations with local and state health departments to refine intent-specific categorizations, improve reporting of patient and facility-level information, and assess patterns of firearm injuries by more detailed geographic characteristics (e.g., county, or rural or urban status) and with consideration of seasonality patterns might further strengthen the use of ED data for firearm injury surveillance.

A comprehensive approach is needed to prevent and respond to firearm injuries and address the social and economic inequities that contribute to the risk for violence. Near real-time ED data can equip public health practitioners, clinicians, researchers, and other partners to quickly identify trends in firearm injuries and develop tailored prevention strategies that fit the needs of their communities. Such strategies might include engaging community and street outreach programs that use trusted community members to de-escalate violent conflicts and connect those with the most need to critical support services, implementing hospital-based programs that intervene with victims of violence, improving community physical environments through vacant lot remediation and greening initiatives, enhancing secure firearm storage to reduce access to means among persons at risk for harming themselves or others, and strengthening social and economic supports for persons and families.^¶¶¶^

**Summary**

**What is already known about this topic?**

During the COVID-19 pandemic, U.S. firearm homicide and suicide rates increased substantially.

**What is added by this report?**

Weekly numbers of firearm injury emergency department (ED) visits began to increase in March 2020 even as the total number of ED visits declined, and sharply increased in late May 2020. Compared with visits during 2019, visits during 2020, 2021, and 2022 were 37%, 36%, and 20% higher, respectively.

**What are the implications for public health practice?**

A comprehensive approach to preventing and responding to firearm injuries is needed, including strategies that engage community and street outreach programs, implement hospital-based violence prevention programs, improve community physical environments, enhance secure storage of firearms, and strengthen social and economic supports.

## Footnotes

* NSSP is a collaboration among CDC, local and state health departments, and federal, academic, and private sector partners. NSSP receives medical record data from approximately 75% of EDs nationwide, although fewer than 50% of facilities from California, Hawaii, Minnesota, and Oklahoma currently participate in NSSP. https://www.cdc.gov/nssp/index.html

†
https://www.federalregister.gov/documents/2020/03/18/2020-05794/declaring-a-national-emergency-concerning-the-novel-coronavirus-disease-covid-19-outbreak

§
https://www.nga.org/coronavirus-reopening-plans/

¶ To reduce artifactual impact from changes in reporting patterns, analyses were restricted to facilities with more consistent reporting of more complete data (coefficient of variation ≤40 and average weekly informative discharge diagnosis ≥75% complete during 2019–2022). https://www.cdc.gov/nssp/dqc/articles/how-data-quality-filters-work.html

** NSSP collects chief complaint, discharge diagnosis, and patient demographics. Diagnosis information is collected using codes from the *International Classification of Diseases, Ninth Revision, Clinical Modification*; *International Classification of Diseases, Tenth Revision, Clinical Modification*; and Systematized Nomenclature of Medicine. Diagnostic codes and free-text keywords were combined using Boolean searches to create categorizations to identify visits for an initial encounter for a firearm injury (including unintentional, intentional self-directed, assault, undetermined intent, legal intervention, and terrorism) and negate subsequent encounters or sequelae.

†† Percent change in visits per week during each surveillance period was calculated as ([mean weekly ED visits for firearm injury during surveillance period − mean weekly ED visits for firearm injury during comparison period] / mean weekly ED visits for firearm injury during comparison period) × 100.

§§ VR = (ED visits for firearm injury [surveillance period] / all ED visits [surveillance period]) / (ED visits for firearm injury [comparison period] / all ED visits [comparison period]). Ratios >1 indicate a higher proportion of ED visits for firearm injury during the surveillance period than the comparison period; ratios <1 indicate a lower proportion during the comparison period than during the surveillance period; 95% CIs that do not include 1 were considered statistically significant.

¶¶ 45 C.F.R. part 46.102(l)(2), 21 C.F.R. part 56; 42 U.S.C. Sect. 241(d); 5 U.S.C. Sect. 552a; 44 U.S.C. Sect. 3501 et seq.

***
https://www.cdc.gov/minorityhealth/racism-disparities/impact-of-racism.html

†††
https://www.cdc.gov/mentalhealth/stress-coping/parental-resources/index.html

§§§
https://www.cdc.gov/nssp/participation-coverage-map.html

¶¶¶
https://www.cdc.gov/violenceprevention/pdf/yv-technicalpackage.pdf
